# Biotechnological Advancements for Improving Floral Attributes in Ornamental Plants

**DOI:** 10.3389/fpls.2017.00530

**Published:** 2017-04-20

**Authors:** Ali Noman, Muhammad Aqeel, Jianming Deng, Noreen Khalid, Tayyaba Sanaullah, He Shuilin

**Affiliations:** ^1^College of Crop Science, Fujian Agriculture and Forestry UniversityFuzhou, China; ^2^Department of Botany, Government College UniversityFaisalabad, Pakistan; ^3^State Key Laboratory of Grassland Agro-Ecosystems, School of Life Science, Lanzhou UniversityLanzhou, China; ^4^Department of Botany, Government College Women University SialkotSialkot, Pakistan; ^5^Department of Botany, University of AgricultureFaisalabad, Pakistan; ^6^National Education Minister, Key Laboratory of Plant Genetic Improvement and Comprehensive Utilization, Fujian Agriculture and Forestry UniversityFuzhou, China

**Keywords:** biotechnology, commercial resource, environment, flower characteristics, horticulture, transgenic plants

## Abstract

Developing new ornamental cultivars with improved floral attributes is a major goal in floriculture. Biotechnological approach together with classical breeding methods has been used to modify floral color, appearance as well as for increasing disease resistance. Transgenic strategies possess immense potential to produce novel flower phenotypes that are not found in nature. Adoption of Genetic engineering has supported the idea of floral trait modification. Ornamental plant attributes like floral color, fragrance, disease resistance, and vase life can be improved by means of genetic manipulation. Therefore, we witness transgenic plant varieties of high aesthetic and commercial value. This review focuses on biotechnological advancements in manipulating key floral traits that contribute in development of diverse ornamental plant lines. Data clearly reveals that regulation of biosynthetic pathways related to characteristics like pigment production, flower morphology and fragrance is both possible and predictable. In spite of their great significance, small number of genetically engineered varieties of ornamental plants has been field tested. Today, novel flower colors production is regarded as chief commercial benefit obtained from transgenic plants. But certain other floral traits are much more important and have high commercial potential. Other than achievements such as novel architecture, modified flower color, etc., very few reports are available regarding successful transformation of other valuable horticultural characteristics. Our review also summarized biotechnological efforts related to enhancement of fragrance and induction of early flowering along with changes in floral anatomy and morphology.

## Introduction

Unequivocally, horticultural industry has been revolutionized due to contribution by ornamental plants. Now a day, diverse ornamental plants are being widely used in home gardening, professional landscaping, and cut flowers as well (Dobres, 2011). Ornamental plant products are globally traded commodities. Due to rising needs, ornamental plant industry requires new plant varieties with elite traits such as improved anatomical attributes, floral color, pigments, stress tolerance, and disease resistance (Chandler and Sanchez, 2012; Azadi et al., 2016). Although we witness extensive employment of classical breeding strategies for developing new plant lines yet limitations and draw backs are also evident i.e., degree of heterozygosity (Shibata, 2008; Da Silva et al., 2011). Since inception of last decade, techniques like genetic engineering (GE), genome editing has been broadly adopted as more feasible methods to deal with intrinsic obstacles of classical techniques (Noman et al., 2016a). Global GM crop cultivation has touched its acme during the last few years (Noman et al., 2016b). Situation can be imagined from the in hand data that reveals GM crop cultivation reached 181.5 Mha in 2014 (Azadi et al., 2016).

Interest as well as contribution of private and government sector toward biotechnology and genetic engineering is increasing day by day. Over the years the main targets were food and feed along with improvements in herbicide and pesticide tolerance. Recently, scientists have also focused on improvement and enhancement of quality attributes for industry (Noman et al., 2016b, 2017; Parisi et al., 2016). The prime benefit in adopting GE is that gene from other species gene pool can be introduced in ornamental plants (Li and Pei, 2006; Chandler and Brugliera, 2011). So it is very possible to introduce genes for disease resistance and stress tolerance in ornamental plant species (Auer, 2008; Kamthan et al., 2016). Similarly, plant characteristics like floral architecture, color, fragrance, resistance to abiotic stress and post-harvest life can be addressed through GE.

This century is considered as the era of bio-economy lead by bioscience and biotechnology. This bio-economy is directly linked to sustainable developments in core areas of agriculture, environment and economy (Huang, 2011). Today, work is going on to produce GM flowers with broad color range and other attributes. Transgenic ornamental plants may become prospective benefit to growers and consumers due to their changed floral appearance, novel colors and improved fragrance (Chandler and Sanchez, 2012). Instead of their immense value, small number of GM ornamental plant varieties have been field tested and released. So far ornamental varieties released in market are mostly the color variant plant varieties e. g., rose (Tanaka and Brugliera, 2013). Therefore, in this review we have highlighted recent advancements in application of GE and biotechnology upon ornamental plants. We have tried to point out developments and requisite attention for far reaching benefits for sustainability of technology and society.

## Why floral traits modifications are of special concern?

Ornamental plants generally possess extraordinarily beautiful and eye catching flowers. Other than aesthetic value, floral traits are crucial for plant survival. These characteristics like shape, shape, fragrance and color have their individual, and collaborative significance. Besides ornamental value, these flowers are utilized in pharmaceutical and other industries. For example, rose plants possess valuable secondary metabolites that are used in production of cosmetics, perfume etc. (Feng et al., 2010). Therefore, floral characteristics have unique importance for plant genetic engineers. Transgenic strategies possess prospective potential for producing innovative flower phenotypes that are not found in nature (Table 1). The genome based modifications for flowers have potential to yield utmost benefits in different aspects. Today, work is going on to produce GM flowers with multiple colors and rest of attributes. Normally, ornamental plants face problems because of troublesome sexual hybridization. This is mainly due to high heterozygosity, high chromosome number, inadequate gene pool, and elevated sterility (Van der Salm et al., 1998; Kim et al., 2006). For example, being alloploid chrysanthemum has chromosome number 36–75 rather than basic chromosome number 9. *Anthurium* sp. have life cycle of about 3 years. Therefore, developing new cultivar may require long time span of about 8–10 years (Azadi et al., 2016). Similarly, most of the carnation lines are self-fertile and unable to produce seeds (Nontaswatsri and Fukai, 2010). Huge genome size in ornamental plants e.g., lilly is a hurdle in mining genomic information (Du et al., 2015). Moreover, other than genome issues, presence or absence of certain metabolites leads to floral changes. One of the reported issues is precocious petal pigmentation and increased sepals pigmentation in *Lisianthus* sp. (Schwinn et al., 2014). Now we will evaluate role of biotechnology and GE for modifications in diverse floral characteristics.

**Table 1:**
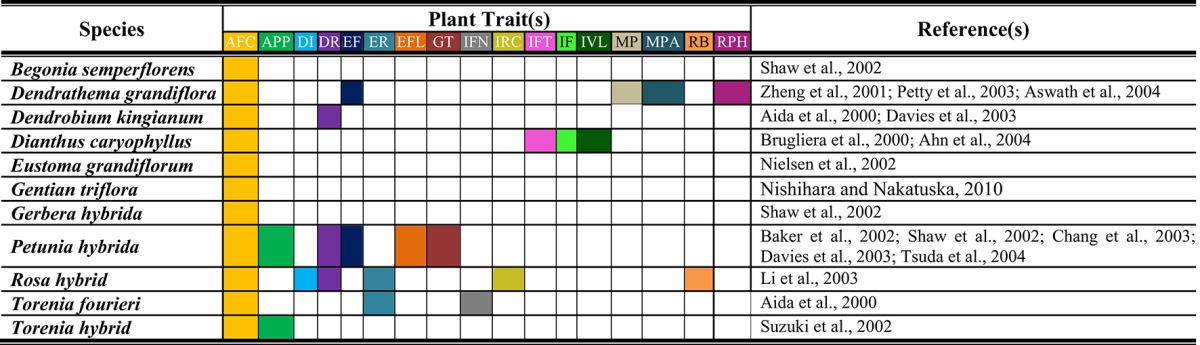
**Successful adoption of biotechnology for modification(s) in various attributes of ornamental plants**.

*AFC, Altered Flower Color; APP, Altered Plant Parts; DI, Disease Improved; DR, Disease resistance; EF, Early Flowering; ER, Enhanced Resistance; EFL, Extended Flower Life; GT, Glyphosate Tolerant; IFN, Increased Flower Number; IRC, Improved Rooting Characteristics; ITF, Improved Fusarium Tolerance; IF, Increased Fragrance; IVL, Increased Vase Life; MP, Modified Phenotype; MPA, Modified Plant Architecture; RB, Reduced Blackspot; RPH, Reduced Plant Height*.

## Plant pigments and flower color

**Figure 1:**
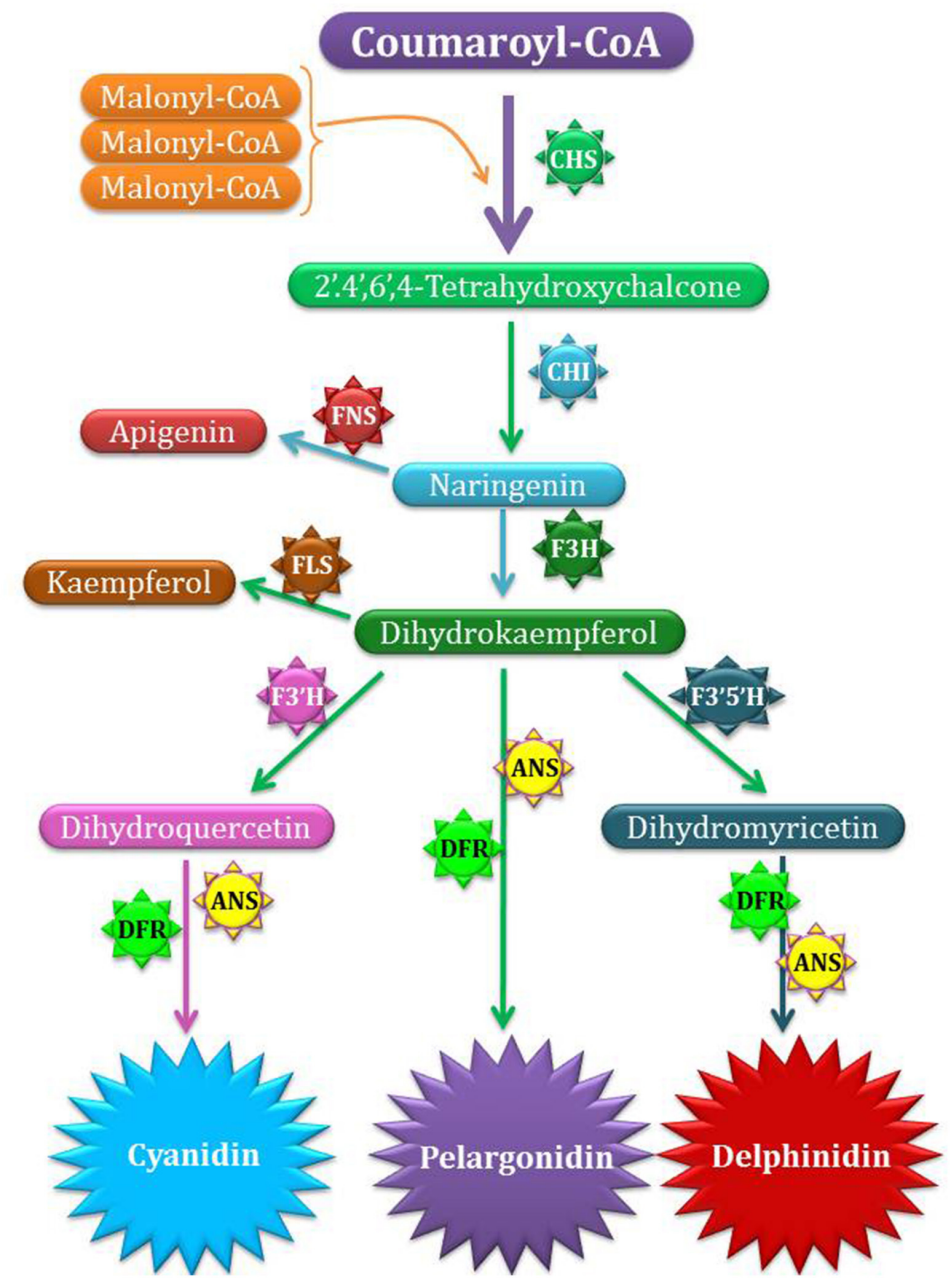
**Biosynthesis of anthocyanidin**. CHS catalyze the formation of Tetrahydroxy calchone. Later on, different enzymes such as CHI, F3H, DFR, ANS catalyze other steps of pigment production. The methyl groups are only added to anthocyanins not to anthocyanidins. The actual pigment color production is not solely dependent upon the enzyme catalyzing reactions but also depends upon other factors. CHS, chalcone synthase; F3H, flavanone 3-hydroxylase; F3′H, flavonoid 3′-hydroxylase; F3′5′H, flavonoid 3′,5′-hydroxylase; DFR, dihydroflavonol 4-reductase; ANS, anthocyanidin synthase; MT, methyltransferase, GT, glucosyltransferase; AT, acyltransferase; FNS, flavone synthase; FLS, flavonol synthase.

Generally, traditional plant breeding strategies are applied to perk up attractiveness as well as effectiveness of ornamental plants. But these strategies face limitations in terms of genes pool and some other characteristics reported in sexually resembling species (Da Silva et al., 2011). During last 20 years, biotechnology has produced innovative and exclusive characters in ornamentals by adopting genes from different plant species (Li and Pei, 2006). Floriculturists and related entrepreneur are always eager to introduce innovations in flower colors. The major pigments responsible for attractiveness of flower colors are anthocyanins, flavonoids, carotenoids, and betalains. Several kinds of anthocyanins are on record (Veitch and Grayer, 2008). These pigments are primarily based upon six anthocyanidins types i.e., cyanidin, delphinidin, peonidin, petunidin, malvidin, and pelargonidin. Three of the described anthocyanidins i.e., delphinidin, cyanidin and pelargonidin are regarded as major types (Figure 1, Table 2). Blue flowers tend to have high level of delphinidin and derivatives while intense red flower color is due to pelargonidin working as anthocyanidin base (Tanaka et al., 2009).

**Table 2:** **Genes involved in synthesis of various enzymes for floral pigment pathways**.

**Sr. No**.	**Plant**	**Gene(s)**	**Enzyme**
1	*Antirrhinum*	*ANS, CHI, CHS, DFR, F3H, F3′H, FNSII, 3GT*	*Anthocyanidin Synthase; Chalcone Flavanone Isomerase; Chalcone Synthase; Dihydroflavonol 4-reductase; Flavanone 3-hydroxylase; Flavonoid 3′ Hydroxylase; Flavone synthase II; Flavonoid-3-O-glucosyltransferase*
2	*Callistephus*	*ANS, F3H, F3′5′H, DFR*	*Anthocyanidin Synthase; Flavanone 3-hydroxylase; Flavonoid 3′,5′-hydroxylase; Dihydroflavonol 4-reductase*
3	*Chrysanthemum*	*CHS, F3H*	*Chalcone Synthase; Flavanone 3-hydroxylase*
4	*Dianthus*	*CHI, CHS, F3H, F3′H, F3′5′H, DFR*	*Chalcone Flavanone Isomerase; Chalcone Synthase; Flavanone 3-hydroxylase; Flavonoid 3′ Hydroxylase; Flavonoid 3′,5′-hydroxylase; Dihydroflavonol 4-reductase*
5	*Eustoma*	*CHI, F3′5′H*	*Chalcone Flavanone Isomerase; Flavonoid 3′,5′-hydroxylase*
6	*Gerbera*	*FNSII, DFR*	*Flavone synthase II; Dihydroflavonol 4-reductase*
7	*Orchid*	*CHS, F3H, DFR*	*Chalcone Synthase; Flavanone 3-hydroxylase; Dihydroflavonol 4-reductase*
8	*Petunia*	*ANS, CHI, FLS, F3′H, F3′5′H, DFR*,	*Anthocyanidin Synthase; Chalcone Flavanone Isomerase; Flavonol synthase; Flavonoid 3′ Hydroxylase; Dihydroflavonol 4-reductase*
9	*Rosa*	*CHS, FLS*	*Chalcone Synthase; Flavonol synthase*
10	*Gentiana*	*3GT*	*Flavonoid-3-O-glucosyltransferase*

Ornamentals like petunia and torenia are considered as more suitable plants for studying floral color modifications produced by genetic engineering. Changes in gene expression were sought out as a base line for production of altered floral colors. Initially, pelargonidin derived anthocyanin production was successfully achieved by maize *Dfr* expression in petunia deficient in *F3*′*5*′*H* and *F3*′*H* (Forkmann and Ruhnau, 1987). We are well aware that varied gene expression for different biosynthetic pathways is leading strategy to achieve flower color variations. For example, variations in genes for flavonoid biosynthesis may generate new colors. In 2007, Katsumoto et al. reported down regulation of *Dfr* gene in hybrid rose and over-expression of the same gene in *Iris hollandica* responsible for delphinidin accumulation in petals. Rose has been specifically emphasized for search of suitable host to achieve blue flower (Yoshida et al., 2009). It has been found that over-expression of voila gene *F3*′*5*′*H* resulted in high delphinidin accumulation and production of blue flower color (Ogata et al., 2005; Katsumoto et al., 2007). Strong role of anthocyanin related genes for rose petals color variation was suggested by Fukuchi-Mizutani et al. (2011). *RhUF3GT2* catalyze flavonol 3-glucosylation in petals which results in accumulation of anthocyanidin 3-glucoside. Zvi et al. (2012) attributed higher production of anthocyanin to introduction of *Arabidopsis* PAP1 transcription factors in transgenic rose. They also confirmed enhanced isoprenoid production in transgenic plants as compared to control plants. Similarly, Chen et al. (2012) informed that white *Chrysanthemum* cultivars e.g., Keikai and Jinba possess major genes for anthocyanins pathways i.e., *Chs, Chi*, and *F3*′*H*.

We can analyze consequences of cross and mutation breeding in ornamental plants. Analysis reveals ultimate consequence as an array of flower colors such as orange, yellow, red, white, and pink. These color changes are directly related to regulation of targeted genes controlling synthesis of pigment precursors. White flowers from different transgenic plants have been obtained by down regulation of genes for anthocyanin production (Tanaka and Ohmiya, 2008). For finding the most suitable promoter for chrysanthemum gene expression, *EF1*α promoter (elongation factor 1α protein) was combined with *GUS* gene for introduction into *C. morifolium* cv. Ramat. Transgenic chrysanthemum plants exhibited high *GUS* expression and petal based transgene expression driven by the 35S CaMV promoter (Aida et al., 2005).

Diverse colors in chrysanthemum are largely resultants of carotenoids and/or red malonylated cyanidin glucosides (Kishimoto et al., 2004). A gene *CmCCD4a* is exclusively expressed in the white chrysanthemum ray petals. This is single dominant gene responsible for inhibition of formation/accumulation of carotenoids in petals (Ohmiya et al., 2009; Yoshioka et al., 2012). So, we may infer from the findings that due to suppression of *CmCCD4a* in white flowers, synthesized carotenoids break down into colorless compound (Ohmiya et al., 2006). Suppression technology like RNAi, co-suppression or antisense mediated silencing have been noticed more helpful in studying flower color variations (Tanaka et al., 2010).

Absence of delphinidin-based anthocyanins in chrysanthemum is chiefly due to deficiency of flavonoid 3′,5-hydroxylase (*F3*′*5*′*H*; Brugliera et al., 2013). That's why we do not observe violet/blue chrysanthemum flowers. Introduction of *F3*′*5*′*H* genes under different promoters control remained successful. Increase in delphinidin accumulation was very prominent under rose chalcone synthase promoter. Success was achieved by observing bluish petals in transgenic plants with higher anthocyanidin content characterized by delphinidin (Yoshida et al., 2009). Silencing of endogenous *F3*′*H* gene with hairpin RNA interference and over-expression of *F3*′*5*′*H* led to production of bluish petal with ~80% delphinidin derived anthocyanins (Brugliera et al., 2013). Combination of various promoters and *F3*′*5*′*H* gene proved enhanced delphinidin-based anthocyanins in transgenic plants.

Anthocyanins, carotenoids or both pigments can be noticed in Lilly sepals (Yamagishi et al., 2012). Azadi et al. (2010) confirmed numerous pigments in the transgenic lilly calli and leaves after transferring some key genes for carotenoid biosynthesis pathway under 35S CaMV. Callus and leaves presented orange color. This is due to presence of keto-carotenoids. In the variety *Lilium* oriental _=_Sorbonne', transient petal transformation by particle bombardment was conducted using three constructs (*Chs pro, HyDfr*, petunia *DifF* using 35S CaMV) possessing *Ph F3*′*5*′*H* (*Phalaenopsis F3*′*5*′*H* gene). Over-expression of *Ph F3*′*5*′*H* resulted in color change from pink to pale purple. A dark purple color was generated by synchronized expression of *Ph F3*′*5*′*H* and *HyDfr* contrary to expression alone. This propose potential role of *HyDfr* gene in facilitating delphinidin production (Qi et al., 2013, 2014). The *GMYB10* over-expression in transgenic gerbera plants considerably improved pigment accumulation and induce cyanidin biogenesis (Laitinen et al., 2008). Non-existence of delphinidin derived anthocyanins in gerbera has produced interest in blue flower production by using genetic engineering. Unfortunately we do not have successful reports for of blue gerbera production yet.

Plants express some genes in cyclical style during the day. This cyclical expression lets them to initiate photoassimilation during sunlight or release scents in the evening when pollinating agents are active. For example development of *Petunia circadia* will facilitate this internal circadian clock to control flower color leading to flower color changing over every 12 h approximately. Similarly, genome modification by means of zinc finger nucleases, CRISPR-Cas systems can help us to attain firmly changed phenotypes (Noman et al., 2016a). For redirecting pigment biosynthesis for achieving desirable color change, not only over-expression of a particular gene taking part in key enzyme synthesis is essential but a selection of appropriate host with suitable genetic background is also mandatory. This selection will be very helpful in reducing antagonism of indigenous pathways with the introduced enzyme or to permit down-regulation of another competing pathway.

## Induction of early flowering

**Figure 2:**
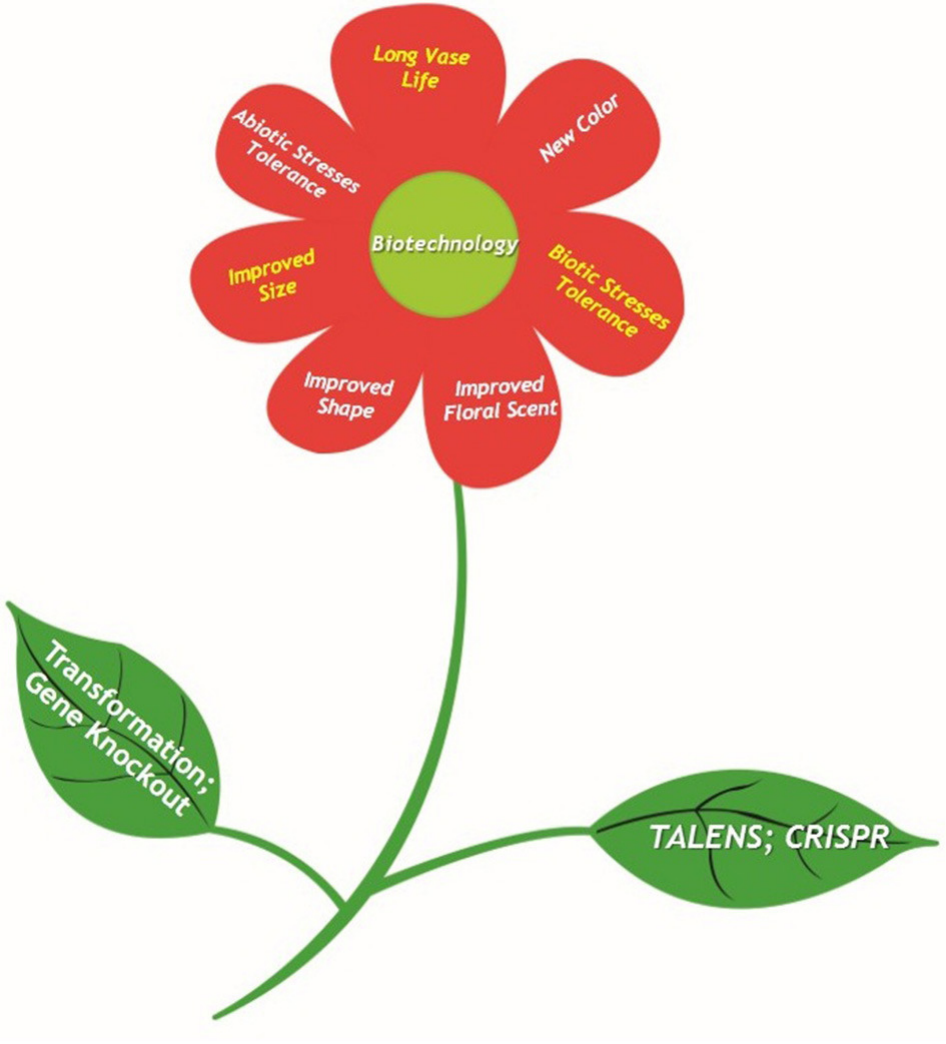
**Benefits from floral traits modification are substantial evidence of successful application of biotechnology**. Advances in genome engineering provide innovation in ornamental plants and crops. These have the potential to circumvent the regulatory concerns raised about GMOs. The competent techniques such as ZFNs, TALENs, and CRISPR-Cas9 enable precise genome engineering by introducing DSBs and NHEJ. The techniques have ability to produce non-transgenic plants for next-generation plant breeding.

Together with synthetic biology, flowers with changing color characteristic have been engineered. Along with other related benefits, the color changing flower is a substantial and striking outcome of biotechnology (Figure 2). It is reflection of ability to outline our world with extensive appeal to all. Flowering time is reckoned as chief determinant for successful commercial plants (Jung and Müller, 2009). Induction of early flowering help plants to cater human needs by bearing more flowers and fruits ultimately. Hence, great economic benefit from ornamental plants can be obtained from establishment of practical floral regulation system. Fundamental studies on flowering time in model plants have described regulatory system of floral transition pathways (Blázquez, 2000). Genetic alterations by means of transforming key flowering associated genes appears to offer valuable advancements for operating floral attributes (Srikanth and Schmid, 2011).

The reduction in flowering time by developing early flowering cultivars or plants able to produce flowers during long days are considerable breeding objectives in ornamental plant breeding (Shulga et al., 2011). The low cost of production make them highly feasible for growers and customers i.e., *Chrysanthemum*. Reports are in hand that comprehensively describe successful gene introduction to produce flowers in comparatively short time. One example is member of MADS box gene family e.g., *AP1* involved in flowering (Litt and Irish, 2003). MADS box genes are indispensable for floral development because they control flowering time as well as floral organ development (Thiruvengadam and Yang, 2009). In transgenic *Chrysanthemum, AP1 o*ver-expression during short-days can start bud initiation 14 days prior to non-transgenic plants. Interestingly, the inserted gene did not exert any change in plant development under long days. The differential gene over-expression during short days is seemingly linked to the plant biochemical changes. Moreover, transgenic flowers displayed prior color configuration and complete inflorescence opening in comparison with non-transgenic plants (Shulga et al., 2011).

Transformation mediated by *Agrobacterium* in *Siningia* sp. supported that exogenous LFY over-expression promotes early flowering (Zheng et al., 2001). Transgenic *Gloxinia* plants with over-expressing or suppressed miRNA159, led to late or early flower appearance respectively. Meanwhile, varied miR159a expression levels resulted in *SsGAMYB* up or down-regulation during flower development. Thus, in transgenic lines transcript level of endogenous *Lfy* as well as *MADS*-box genes is changed. So it was established that miR159-mediated *GAMYB* expression also play crucial role in controlling flowering time period in ornamentals (Li et al., 2013). This will be helpful in establishing a practical regulation system that will facilitate flowering to be accelerated or delayed proportional to the demand. Fu et al. (2012) have demonstrated that rice miR156 over-expression amplify biomass and increment number of tillers in *Panicum*. Hence, it appears to be a competent strategy for boosting or curtailing target genes activity via transformation of conserved miRNA in different plant species.

In *Chrysanthemum seticuspe, CsFTL3* gene (Flowering Locus T like paralog) has been found to work as photoperiodic flowering regulator. Over-expression of this gene can induce flowering under long day conditions in *Chrysanthemum* (Oda et al., 2012). RNA sequencing has been adopted to illustrate quantitative effects of bulb vernalization on flowering and facilitates gene expression in the meristems. Investigations involving lilly bulbs subjected to cold treatment revealed many feasible candidate genes involved in vernalization (Huang et al., 2014; Villacorta-Martin et al., 2015).

In *Glebra*, transformation of *GSQUA*2 gene accelerated flowering (Ruokolainen et al., 2010). In 2011, Ruokolainen et al. detected three paralogs of *At*-*SOC1*. Over-expressing *Gh-SOC1* resulted in fractional loss of floral distinctiveness. This over-expression caused decrease in epidermal cell size in ray flower petals along with shape modification but flowering time remained uninfluenced. Conversely, *Gh-SOC1* down regulation failed to produce any considerable phenotypic alteration in transgenic plants (Ruokolainen et al., 2011). In *Lisianthus* sp. transformation of *OMADS1* showed significant reduction in flowering time as well as increased no. of flowers in comparison to non-transformed ones.

Of the reported genes dealing with flowering pathways, *FT* (*Flowering locus* T) is chief integrator of several flowering genes that respond to different signals such as light, temperature etc. (Fornara et al., 2010). *FT* supports flowering by up-regulating flowering genes such as *AP1, Lfy*, and *SOC1*. Over-expression of *VcFT*-Aurora in the transgenic blueberry supported early and continuous flowering in both *in vitro* shoots and greenhouse plants. Additionally, all transgenic *VcFT*-Aurora plants exhibited dwarf phenotype (Gao et al., 2016). The end result of *VcFT-OX* is differential expression of 110 pathway genes for five phytohormones. This connection between phytohormones pathway genes and *VcFT* implies a probable role of phytohormones as signals regulating plant growth and development.

Mostly, the knowledge regarding flowering time regulation is applied by means of gene over-expression or suppression of gene activity in crops (Jung and Müller, 2009). However, the research has not met full commercial execution. Due to multiple reasons, we are expecting to see wide range transgenic flowers in market. Less advancement in ornamentals can be observed in case of targeted genetic change for flowering time. The restricted access is seemingly because of relative unidentified genomic information in most of the commercial plant species. Hence, it is mandatory to search an appropriate approach for generating fresh varieties with changed flowering conduct in commercial plants. A substantial sum of try and error is certainly required to attain an agreeable final result. Analysis of in hand data pinpoints genetic modifications in ornamental plants as realistic and flourishing scheme both in scientific and commercial perspective.

## Floral anatomy and morphology

Customers demand for purposeful and distinctive horticultural plants urge scientists for development of novel cultivars, their large scale production and circulation as well. Ornamental plants are cultivated with rationale of beautifying, embellishing, or improving human environments irrespective of indoor or outdoor. Novel floral figure in ornamental plants is essential for high market value. Up till now, molecular events for developing flower pattern in the meristem have remained clandestine. Primarily this is due to significant inflorescence variations among plant species. Majority of these plant types are not cross-compatible with each other. Therefore, we agree upon the decision that conventional cross-breeding strategy cannot be applicable to point out the genes responsible for flowering patterns.

The diverse gene behaviors and their differential expressions reflect diversity in plant responses. In the start of this century, tobacco *phytochrome b1* gene over-expression in chrysanthemum led to production of small sized plants but with larger branch angles (Zheng et al., 2001). Later, decrease in chrysanthemum plant height was achieved by introducing *Arabidopsis* GA insensitive gene (Petty et al., 2003). Furthermore, Aida et al. (2008) demonstrated transfer of *CAG* gene in antisense orientation into *C. morifolium*. They revealed that *CAG* gene suppression alters gynoecium and androecium to corolla-like tissues. However, the alteration pace was very small and simply changed the phenotype for flower-shape. It has been reported that *Ls*-like antisense gene expression in transgenic *Dendranthema grandiflorum* result in drop of axillary branching (Han et al., 2007). *PttKN1*ectopic expression in *D. caryophyllus* L. resulted in pleiotropic morphological wavering inclusive of phyllotaxis modifications (Meng et al., 2009).

GRAS TF restrains lateral branch production. Transformation of *DgLsL* gene with vector pCAMBIA1301-sense and antisense *DgLsL* was attributed to abundant branches in chrysanthemum. Conversely, branching was lessened in antisense transformants (Jiang et al., 2010). Collective silencing of *DmCPD* and *DmGA20ox* genes is a feasible strategy to create dwarf chrysanthemum varieties (Xie et al., 2015). It has also been proved that *D27* gene mutants (gene involved in strigolactone biosynthesis) reveal high tillering with dwarf phenotypic appearance (Lin et al., 2009). Cloned *DgD27* from *D. grandiflorum* expression in *Arabidopsis* put forward a new approach for development of chrysanthemum cultivars with reduced number of tillers (Wen et al., 2016). Similarly, *GSQUA*2 over-expressing transgenic *Gerbera* plants presented vivid vegetative alterations like elongation of vegetative axis. However, these plants were found to be infection susceptible with very limited root formation and tiny inflorescence (Ruokolainen et al., 2010). Thiruvengadam and Yang (2009) demonstrated that transgenic plants with 35S::*L MADS1-M* gene from lily produced more flowers from leafy branches in comparison to wild types. Moreover, transgenic *Lisianthus* flowers displayed a change of floral structure. Transgenic plants were characterized with conversion of petal second whorl into sepal like structures and deformation of third whorl stamens. Of note, transgenic carnations harboring *rol C* gene with promoter 35S CaMV demonstrated higher stem cutting yield and flowering stems and root organogenesis (Zuker et al., 2001; Casanova et al., 2004). So we can attribute such gene expression as solid evidence of their function for endorsing changes in floral shape and structure Conclusively, molecular breeding methods allow critical assessment of biological processes for floral changes and help scientific community as well as public to put GE of ornamental plants as deep insight regarding its effectiveness in plant breeding.

## Fragrance engineering

Floral scents have vital function in plants reproductive process and possess substantial economic significance. It essentially improves aesthetic characteristics of ornamental plants. Many floral scent volatiles fall into the category of terpenoid, phenylpropanoid/benzenoid, and aromatic amino acid (Oliva et al., 2015). Flowers produce a great range of specific metabolites like fragrant volatiles to attract pollinators, hormones to stimulate or repress signaling cascades and fragrant volatiles for protection against herbivores or pathogens (Baldwin, 2010; Dudareva et al., 2013). The array of particular metabolites synthesized by flowers of different plants is wide-ranging (Muhlemann et al., 2012; Zvi et al., 2012). On the other hand, different flower specific metabolites are actually produced in low amounts. Thus, their detection and characterization is cumbersome task. Therefore, enhanced production of floral specific metabolites may contribute in detection, isolation, and identification of compounds and improvement of flower properties like fragrance and pigmentation. Although the floral scent biochemistry is relatively new field yet during last decade researchers identified several scent controlling genes. Not all but a number of scent genes encode enzymes that directly catalyze the formation of volatile compounds. The manipulation of scent genes through genetic engineering has revealed success in adoption of this technology for amplifying floral scent potential.

For induction of fragrance in petals, *C. breweri BEAT* gene (benzyl alcohol acetyl transferase for benzyl acetate production) was introduced in *Lisianthus* (Aranovich et al., 2007). Recorded observations affirmed volatile compounds production including benzyl acetate among transgenic leaves and flowers upon feeding with alcoholic substrate. From the results, we can infer that alcoholic substrates are mandatory for fragrance induction by GE in lisianthus cut flowers. The innovative transcriptomic profiling offer substantial source for plant functional genomics and provide us deep insight in biological processes for petals development in *H. coronarium*. These data helps to elucidate the molecular mechanism of floral scent genesis and its regulation in monocotyledons (Yue et al., 2015).

Production of specialized metabolites for scent biosynthesis is not solely dependent upon enzymatic actions and greatly relies upon several TFs. In recent years, transcriptional regulation of fragrance biosynthesis pathways has been deeply studied (Muhlemann et al., 2012) that implies crucial roles of different transcriptional factors in controlling scent emission (Colquhoun and Clark, 2011). In spite of immense value, few TFs involved in fragrance emission regulation have been identified. From petunia petals, exclusively expressed *ODORNT1* (ODO1) has been found to regulate shikimate pathway (Verdonk et al., 2005). *ODO1* was also reportedly involved in promoter activation of plasma membrane based ABC transporter of unknown function (Van Moerkercke et al., 2012). Petunia *EOBI* (emission of benezoids 1) is R2-R3 type flower specific transcription factor that acts upstream of *ODO1* and downstream of EOBII. Silencing of this *EOBI* expression caused down regulation of several shikimate pathway and fragrance related genes (Spitzer-Rimon et al., 2012).

TFs regulating phenyl propanoid/benezoid pathways have been detected and characterized but terpenoids pathway transcriptional regulation is still obscure. Few years back, in Arabidopsis inflorescence, *MYC2* helix loop helix TF was detected for expression of two *TPS11* and *TPS21*sesquiterpene synthase genes (Hong et al., 2012). Other than identification and characterization of different TFs, master regulators which control diverse volatile compound production and different associated metabolic pathways are yet to be discovered. Recent progress in identifying the genes and their product enzymes taking part in volatile compounds biosynthesis have declared this metabolic engineering extremely practicable. Noteworthy successes are on record in improving plant defense and enhancing scent and aroma value of flowers and fruits (Table 3). The result of discussed research indicates that the GE for changing flower scents has significant potential. But, research outcomes also expose the drawbacks resulting from our insufficient knowledge of the scents metabolic processes and their regulation.

**Table 3:** **Role of different genes for improved phenotypes in GM ornamental plants**.

**Source**	**Gene**	**Result(s)**	**Reference(s)**
*Agrobacterium rhizogenes*	*RolC*	Dwarfed Pelargoniums and Petunias	Boase et al., 2004
*Agrobacterium tumefaciens*	*ipT*	Increased branching and reduced internode length in Chrysanthemum	Khodakovskaya et al., 2009
*Arabidopsis*	*Asl38/lbd41*	Flowers turned into multiple column patterns in *Celosia cristata*	Meng et al., 2009
	*Ft*	Activate the floral identity genes; promotes flowering in appropriate conditions	Jung and Müller, 2009
*Carnation/apple*	*ACO/ACS-coding genes*	Increased vase life in carnation	Zuker et al., 2001
*Chrysanthemum*	*Ls*	Less branching in Chrysanthemum	Han et al., 2007; Jiang et al., 2010
	*AP1*	Speeds up time to flowering in Chrysanthemum	Jiang et al., 2010
	*ERS1*	Mutated gene slows down yellowing of leaves in *Chrysanthemum*	Narumi et al., 2005
*Gentian*	*Chs*	Gene silencing produces white flowers in *Gentian*	Nishihara and Nakatuska, 2011
	*Ans*	Gene silencing produces pale blue flowers in *Gentian*	Nakatsuka et al., 2008
*Lotus*	*CrtW*	Overexpression changes petal color from light yellow to deep yellow or orange in Lotus	Suzuki et al., 2002
*Orchid/Lily*	*MADS-Box*	Ectopic expression changes the second round of petals into calyx in orchids and lilies	Thiruvengadam and Yang, 2009
*Petunia/Pansy*	*F3′-5′H*	Overexpression produces blue flowers in combination with a silenced dfr gene in carnation (Petunia) and Roses (Pansy)	Katsumoto et al., 2007

## Abiotic and biotic stress tolerance

Plant growth and productivity are severely affected by abiotic and biotic stresses (Ali et al., 2016; Islam et al., 2016; Noman and Aqeel, 2017). Due to inadequate number of resistance genes, breeding for stress resistance/tolerance in ornamental plants is difficult (Azadi et al., 2016). During last few years, use of biotechnological strategies for conferring resistance against abiotic and biotic stresses e.g., drought, pathogen attack have gained attention (Table 4). The critical appraisal of transgenic ornamental plants indicates resistance to biotic stresses in comparison to the wild plants (Chandler and Sanchez, 2012; Teixeira da Silva et al., 2013). Fungal, viral, or bacterial pathogens sternly affect plants by decreasing plant growth and yields (Islam et al., 2016, 2017) inclusive of reduced quality of ornamental products. In 2002, Dohm et al., 2002 reported transgenic roses possessing antifungal genes i.e., *class II chitinase* and type I RIP (ribosome inhibiting protein). In rose, resistance to powdery mildew disease was improved by introduction of rice-chitinase gene (Pourhosseini et al., 2013). Three N-methyltransferase genes e.g., *CaXMT1, CaMXMT1*, and *CaDXMT1* were introduced in *D. grandiflorum* cv. Shinba. The observations confirmed high resistance to *Botrytis cinerea* (Kim et al., 2011). In chrysanthemum, transfer of *PGIP* gene (polygalacturonase-inhibiting protein) from *Prunus mumei* reduced disease index and occurrence of *Alternaria* leaf spot (Miao et al., 2010). Similarly, *chi*II endorsed improved resistance to septoria leaf spot disease in transgenic chrysanthemum (Sen et al., 2013). De Cáceres González et al. (2015) have produced transgenic *L. longifolium* cv. Star Gazer with high resistance against *B. cinerea*. The ectopic over-expression of the RCH10 gene (*Rice Chitinase 10*) under 35S CaMV promoter was found positively correlated with high resistance to *Botrytis*. Recently, several SNP markers have been identified for additional linkage mapping along with other transcripts that might have involvement in *Botrytis* resistance pathways (Fu et al., 2016).

**Table 4:** **Recent biotechnological advances and milestones for improving ornamental plants**.

**Sr. No**.	**Category**	**Source**	**Transferred to**	**Gene**	**Function**	**References**
1	Abiotic Stress Tolerance	*Arabidopsis thaliana*	*Rosa hybrida*	*PAP1* transcription factor	Enhance production of anthocyanin and also eugenol (phenylpropanoid compound) accumulation increases	Zvi et al., 2012
2		*Medicago truncatula*	*Rosa chinensis Jacq*.	*MtDREB1C*	Freezing stress tolerance enhancement	Chen et al., 2010
3		*Dendranthema grandiflora*	*Chrysanthemum morifolium_=_Sei-Marine'*	*mDG-ERS1 (etr1-4)*	Reduces ethylene sensitivity	Narumi et al., 2005
4		*Arabidopsis thaliana*	*Dendranthema grandiflorum _=_Fall Colour'*	*AtDREB1A*	Heat tolerance improvement	Hong et al., 2009
5		*Dendranthema vestitum*	*Chrysenthamum*	*DvDREB2A*	Enhances tolerance to heat & Water stresses	Liu et al., 2008
6		*Prunus mume*	*chrysanthemum*	*PGIP*	Reduces disease index & increases resistance to *Alternaria* leaf spot disease	Miao et al., 2010
7	Biotic Stress Resistance	*Oryza sativa*	*Chrysanthemum*	*Rice chitinase* (*chiII*)	Creates *Septoria* leaf spot disease Resistance	Sen et al., 2013
8		*Oryza sativa*	*Lilium oriental _=_Star Gazer'*	*Rice Chitinase 10* (RCH10)	Tolerance to *Botrytis cinerea*	De Cáceres González et al., 2015
9		*Oryza sativa*	*Rosa*	*Rice*-*chitinase* gene	Improves tolerance to *powdery mildew*	Pourhosseini et al., 2013
10		*Avena sativa*	*Dianthus caryophyllus*	*Oat thionin*	resistant to *Burkholderia* infections	Shirasawa-Seo et al., 2002
11		*Oryza sativa*	*Chrysenthamum*	*RCC2*	Impervious to *gray mold*	Takatsu et al., 1999
12		*Xanthomonas oryzae* cv. *oryzae*	*Chrysanthemum morifolium*	*hpaGxoo* gene	Increases resistance to *Alternaria* leaf spot disease	Xu et al., 2010
13		*Prunus mumei*	*Chrysenthamum*	*PGIP*	Increases resistance to *Alternaria* leaf spot disease	Yu et al., 2010
14		*B. thuringiensis*	*Chrysanthemum*	*cry1Ab*	Induces insect resistance	Shinoyama et al., 2008
15	Vegetative Character(s) Improvement	*Nicotiana tabacum*	*Chrysanthemum “Iridon”*	*Tobacco phytochrome b1*	Shorter plants with larger branch angles	Zheng et al., 2001
16		*Arabidopsis thaliana*	*Chrysanthemum morifolium*	*A. thaliana GA insensitive gene*	Reduction in plant height in was achieved	Petty et al., 2003
17		*Dendranthema grandiflorum*	*Chrysanthemum morifolium*	*DgLsL*	Produced profuse lateral branches	Jiang et al., 2010
18		*Cucumis melo*	*Chrysanthemum*	*CmETR1/H69A*	Significantly Reduces fertility for both male & female	Shinoyama et al., 2012
19		*Gerbera hybrida*	*Gerbera hybrida*	*Overexpression of GSQUA*2	Rapid vegetative alteration like dwarfism and vegetative axis elongation	Ruokolainen et al., 2010
20		*Clarkia breweri*	*Eustoma grandiflorum*	*Clarkia breweri* gene (*BEAT*)	Induces fragrances in petals	Aranovich et al., 2007
21		*Orchid*	*Eustoma grandiflorum*	*OMADS1*	Alteration in flowering time	Thiruvengadam and Yang, 2009
22		*Clarkia breweri*	*Dianthus caryophyllus*	*linalool synthase*	Fragrance production	Lavy et al., 2002
23		*Arabidopsis*	*chrysanthemum*	*TCP3* chimeric repressor,	Production of fringed leaves	Narumi et al., 2011
24		*Cucumus melo*	*Chrysanthemum*	*CmETR1/H69A*	Reduction in male and female sterility	Shinoyama et al., 2012
25		*Petunia*	*Lilium*	*DifF*	Production of purple colored petal cells	Azadi et al., 2016

Environmental conditions play very important role in survival of plants. Presence of pathogens and their infection dissemination is directly proportional to prevailing environmental conditions. In 2008, Clarke et al. (Clarke et al., 2008) developed GM *Euphorbia pulcherrima* with enhanced resistance to virus. Different genes from various sources have been validated for their crucial role in plant life during stress conditions. Targets of these biotic or abiotic stress-related genes also function in various cellular responses and metabolic processes e.g., transcriptional regulation, cell proliferation which indicate the variety of gene functions to tackle abiotic stress. For example, in transgenic chrysanthemum, over-expressing *hpaGxo*o gene increased resistance to *Alternaria tenuissima* (Xu et al., 2011). Agrobacterium mediated transformation of coat protein gene (cp) inducted resistance against CMV (Cucumber mosaic virus). Integration of cp gene in *C. morifolium* genome enhanced acclimatization percentage of transgenic plants displaying no or least CMV symptoms (Kumar et al., 2012). Transgenic *Grebra* plants harboring Nucleoprotein gene (*N*-gene) did not display TSWV (Tomato spotted wilt virus) symptoms (Korbin, 2006). Similar to this, transformation of glagiolus plants with sense or antisense orientation CP gene survived well under BYMV attack (bean yellow mosaic virus; Kamo et al., 2005).

Review of literature affirmed that expression of *cry1Ab* gene in chrysanthemum is strongly related to insect resistance i.e., *Spodoptera litura* (Shinoyama et al., 2012). Agrobacterium mediated transformation of LLA gene (*Lycoris Longituba agglutinin)* incremented resistance to aphids in transgenic chrysanthemum plants (He et al., 2009). Stable expression of different genes in ornamental plants like lilies has shown promising results in outdoor or green house conditions. Genes such as *bar, nptII* has been reported highly expressed in transgenic Lily plants under varying environmental conditions (Kamo, 2014). Transformation of *OcI*Δ*D86* into *Lily* cv. Nellie White exhibited comparatively high resistant to an endoparasite, *Pratylenchus penetrans* causing RLN (root lesion disease). *OcI*Δ*D86* expression inhibited parasitic effects of *Pratylenchus* by 75% in transgenic plants as compared to their wild relatives (Vieira et al., 2015; Table 4).

Saline soil, nutrient acidity, nutrients imbalance, water scarcity are major constraints that severely cut off plant yield, and other attributes (Noman et al., 2015; Zafar et al., 2016). Different strategies such as conventional plant breeding and genetic engineering are in use for developing abiotic stress tolerant varieties (Noman et al., 2016b). Several transcription factors e.g., ZIP, WRKY, NAC, and their products are crucial in plant response to stress conditions like drought, high temperature etc. (Vinocur and Altman, 2005). Ornamental plants also exhibit different responses upon exposure to abiotic stresses. For example, *Rosa* sp. is very susceptible to cold stress. *R. chinensis* Jacq. was transformed with *Medicago* gene *MtDREB1C*. Transgenic plants performed well under freezing temperature and hence proved improvement in tolerance to low temperature (Chen et al., 2010). It was proposed that frost tolerance in petunia may be enhanced by transforming *AtCBF3* gene (Warner, 2011). Hong et al. (2006a,b) developed low temperature resistant chrysanthemum lines by transformation of *AtDREB1A* gene. They noticed great increase in transgenic seedling and plant growth in winter. Two constructs i.e., 35S:*DREB1A* and *rd29A*:*DREB1A* were integrated into chrysanthemum genome for enhancing tolerance to salinity and water deficiency. The *rd29A*:*DREB1A* transgenic plants showed more tolerance in comparison with 35S:*DREB1A* transgenic plants (Hong et al., 2006a). Furthermore, *AtDREB1A* over-expression in *D. grandiflorum* produced sturdy heat tolerance. Upon exposure to 45°C for 36 h, wild and transgenic plants exhibited a marked difference in survival rate e.g., 20 and 70%, respectively (Hong et al., 2009). Salt stress tolerance of *chrysanthemum* cv. Jinba was enhanced with *CcSOS1*constitutive expression encoding plasma Na+/H+ antiporter (An et al., 2014; Song et al., 2014). Ascorbic acid (AsA) has very important role in abiotic stress tolerance (Noman et al., 2015). *GLDH* (L-galactono-1, 4-lactone dehydrogenase) catalyzes the oxidation of L-galactose to AsA (Agius et al., 2003). *pCAMBIA* vector harboring *GLDH* from apple was used for transformation of *L. davidii* var. unicolor. The results highlighted that *GLDH* over expression in transgenic plants, considerably amplified AsA production and endorsed resistance against abiotic stresses (Shi et al., 2012). In the light of above quoted examples, we are convinced that genetic engineering offers unprecedented arena for plant trait improvement. Particularly in ornamental plants, attributes like environmental stress tolerance, disease resistance can be retained in transgenic cultivars coupled with improved product range.

## Shelf life enhancement

One of the daunting challenges for researchers is to increase shelf life of ornamental plant products coupled with maintained characteristics such as aroma, taste, etc. (Kamthan et al., 2016). Transgenic ornamental plants have potential to enhance leaf and flower longevity. Generally, cut flowers are treated with different kinds of chemicals for increasing their shelf life (Teixeira da Silva et al., 2013). With special reference to cut flowers, improved shelf life is decisive attribute and is of particular significance for breeders. To achieve the target of enhanced vase life, different biotechnological techniques have been used (Matas et al., 2009). Decreased autocatalytic ethylene synthesis by suppression of genes for ethylene production pathway such as *ACO* (ACC oxidase) or *ACS* (1-aminocyclopropane-1-carboxylicacid synthase) has been found effective to improve fruit vase life (Hamilton and Baulcombe, 1999). Transgenic carnation plants exhibiting delayed petal senescence have been developed by silencing the *ACO* gene that down regulate ethylene synthesis (Savin et al., 1995). Bovy et al. (1999) developed transgenic carnations harboring *Arabidopsis etr1-1* gene. Vase life of transgenic flowers was incremented 3 times in comparison with control plant.

Increased shelf life can be achieved by maintaining resistance to ethylene or by the inhibition of ethylene biosynthesis genes. Success has been witnessed in *Oncidium* and *Odontoglossum* by mutating ethylene receptor gene (Raffeiner et al., 2009). Different research groups have conducted experiments involving *ACS* or *ACO* genes in antisense fashion for increasing the shelf life of ornamental plant products (Bapat et al., 2010; Litz and Padilla, 2012). Transcriptional regulators such as *ERF*s (Ethylene response factors) modulate ethylene-induced fruit ripening. In transgenic *Lycopersicum esculentum*, antisense *LeERF*1 fruits exhibited more vase-life contrary to the wild-type plants (Li et al., 2007). Vase life of transgenic pot plants has been maintained by developing ethylene sensitive pot plant species with lessened ethylene sensitivity (Sanikhani et al., 2008; Milbus et al., 2009). Agrobacterium mediated transformation of *PSAG12-ipt* in Rose cultivar “Linda” augmented the *ipt* expression level in transgenic plants upon exposure to darkness and high level of exogenous ethylene. Moreover, chlorophyll amount was considerably increased (Zakizadeh et al., 2013).

Normally, leaves of cut flowers show yellowness, even before the start of senescence. On the other hand, exposure to ethylene can also speeds up yellowing. This yellowing of leaves is a feature of senescence. This destroys flower attractiveness, diminishes quality and shortens vase life. Consequently, transgenic plants with reduced ethylene sensitivity i.e., chrysanthemum is anticipated to possess improved vase life (Satoh et al., 2006). In transgenic *D. grandiflorum*, reduced leaf senescence has been proved very useful (Satoh et al., 2008). Similarly, transformation of *etr1-1* gene impeded senescence in petunia. But unfortunately this senescence reduction was coupled with curtailed rooting of cuttings that is unacceptable in market (Gubrium et al., 2000). This deficiency highlights need for a more tissue specific expression gene of genes regulating some crucial processes (Chandler and Sanchez, 2012). Additionally, the endogenous production and distribution of plant growth regulators such as IAA, iPA, and ABA during the course of flower induction and initiation may facilitate us to better plan the harvest timing for maximum flower uniformity and the quality of ornamentals (Jiang et al., 2010; Teixeira da Silva et al., 2013).

## Concluding remarks

Worldwide increase in economic worth of ornamental plants is somehow a result of promising prospects of gene transformation. Novel transgenic ornamental plants may therefore provide prospective benefits to growers and consumers by generating diverse floral appearance, novel colors, and improved fragrance. Taking aid from in hand data, we recommend that genetic modification of ornamental plants is very pragmatic and successful scheme both in scientific and commercial perspective. We should not be worried about acceptance of transgenic flowers in the market. Very luckily, consumers have shown their satisfaction over genetically modified flowers e.g., rose, carnation. Transgenic ornamental varieties have been proved successful during vegetative propagation and have not shown any undesired effect on environment or on the health of handler. Several exploitable traits of ornamental plants can be particularly associated with secondary metabolites production such as phytoceuticals. Development of molecular markers and complete genome sequences of ornamental plants will lead to discovery of new genes and related pathways. This will momentously contribute in production of new transgenic varieties of ornamental plants. Furthermore, application of novel techniques like ZFNs, TALENs, and CRISPR has substantial potential to facilitate floriculture industry by targeted genomic modifications. Despite sound scientific prospects of transgenic flowers, economic and regulatory barriers have hampered the expansion in commercialization of GM ornamentals. Time has come to address regulatory obstacles for commercial release of GM plants including flowers. In absence of internationally apt and approved process for regulation of GM products, release of ornamental products will remain very difficult due to expenditures and capability needed for commercial development. To alleviate such inconvenience the regulatory requisites for non-food plants like ornamentals ought to be decreased.

## Author contributions

AN has collected research data and compiled manuscript. MA has made all figures and tables. NK and TS have compiled table and corrected the references as well as DOI. JD and HS have critically read this manuscript and suggested for improvement.

### Conflict of interest statement

The authors declare that the research was conducted in the absence of any commercial or financial relationships that could be construed as a potential conflict of interest.
